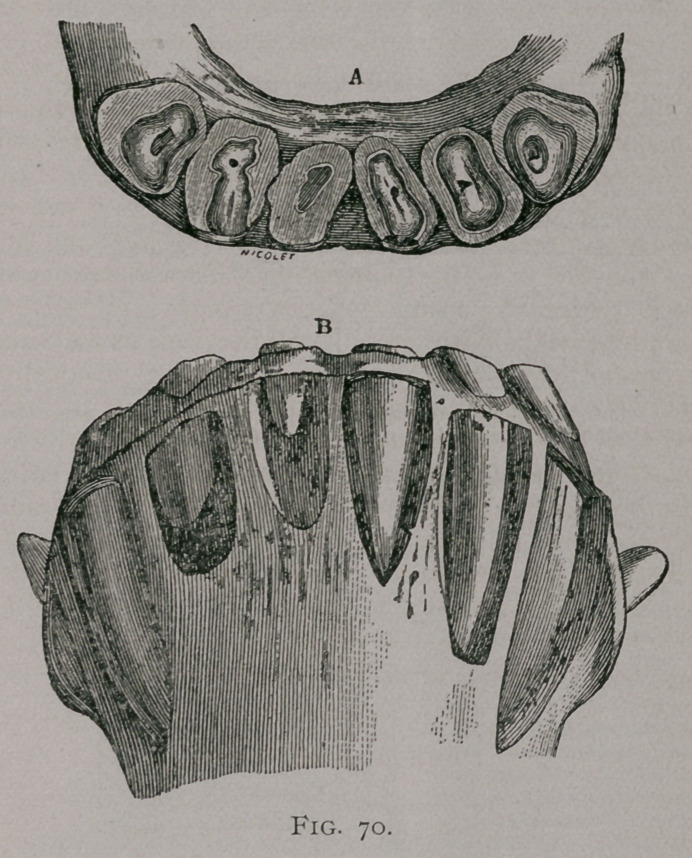# Age of the Horse, Ox, Dog, and Other Domesticated Animals

**Published:** 1891-05

**Authors:** R. S. Huidekoper

**Affiliations:** Vet.


### AGE OF THE HORSE, OX, DOG, AND OTHER DOMESTICATED ANIMALS.

BY R. S. HUIDEKOPER, M.D., VET.

[*Continued from page 180.*]

### IRREGULARITIES FROM FAULT OF LENGTH, OR EXCESS OF SIZE, OF ONE OF THE JAWS.

Deficiency in length of either of the jaws is rare in the horse, but sometimes occurs. The simplest form is where there is a slight projection of the lower jaw, (prognathism in man), in this there is excessive wearing of the lower incisors which complicates the determination of age. A more serious abnormality is deficiency in length in the lower jaw, (brachygnathism in man), which is followed by deformity of the intermaxillary bones; these curve down and interfere greatly with the prehension of food, especially at pasture.

In prognathism the lower table may project for a variable distance beyond the upper jaw, but it is rare that it extends beyond to any marked degree, and while it causes the teeth to wear away rapidly, there is not often any alteration in the incidence of the jaws. In brachygnathism, the difference in length of the two jaws may be very great, in some cases so great, that the upper jaw completely over-reaches the lower one, and by bending of the intermaxillary bones, the upper teeth drop over the anterior face of the lower teeth, while the latter point their tables directly into the hard palate; in other cases the posterior face of the upper incisors wear against the anterior face of the lower incisors until both become beveled into sharp wedges.

### EXCESS OF WIDTH OF THE UPPER INCISIVE ARCH.

This condition is normal in old horses, without inconveniencing them, it sometimes occurs in young horses, in which the irregular wearing against the upper teeth produces marked notching, and sometimes interferes with the prehension of food; in one case, in which the excess of breadth of the upper incisive arch was due to the presence of supernumerary corner teeth, giving the arch eight teeth in all, the deformity was very marked.

### IRREGULARITIES BY EXCESS OR FAULT OF USE.

As a general thing the incisive teeth have the same length in their free portion, although the whole tooth is constantly shortening each year, from the wearing away of its table, this even length is maintained by the roots being constantly forced from the alveolar cavities, and becoming a part of the free portion; transverse lines, filed at definite points on the anterior surface of the incisors, will be seen to approach the table and disappear one after another. Experiments by Pessina* showed that in common horses the annual wearing is about 4 1-2 millimeters while in thoroughbreds it is only about 3 millimeters; experiments by Bouley verified those of Pessina.

Girard, after numerous investigations, determined that the free portions of the incisors, from the gum to the table, averages 15 millimeters; this is subject to some variation, but normal pinchers rarely vary from 18 millimeters in length, intermediate teeth beyond 15 millimeters, while corner teeth are about 13 millimeters; variation in the length of the incisors may be from excess of length, or from deficiency of length.

### EXCESS OF LENGTH.

This anomaly occurs in several ways, either occurring in both jaws, or in the teeth of the upper jaw alone, or at times only in certain teeth of one or the other jaw.

### EXCESS OF LENGTH OF BOTH JAWS.

When the teeth of both jaws are too long, there is a general tendency for them to become parallel, that is to approach a horizontal direction, but their free extremities are diverged like the

* Pessina, Sul modo di conoscere dai denti l'eta dei cavelli. Traduit de l'allemand par Luggi Ferreri et revu par Giuseppe Antonio Gross; Milano, 1831, p. 24 et pl. IX.

ribs of a fan and the wearing surface, or table, shows the character of the age of the horse; flattened in front to behind, they do not tend to take an oval form, the central enamel occupies a large portion of the dental table; there is often a little external cavity in the inferior corner teeth, the excess of length of the crown of the tooth is not in proportion to that of the root, the teeth are less solidly fixed in their alveolar cavities, and are subject to fracture from moderate violence, on account of the great leverage of the lengthened crown. These teeth on their table apparently show an age, which is sometimes very deceptive. To determine the exact age, it is necessary to shorten, by the imagination, the elongated teeth; determine by a close examination what would be the form of the tables of the teeth, were they cut off to their proper length; what would be the form and position of the cups and dental stars in such a shortened tooth; and give to the animal the age, which the alterations indicate.

FIG. 69.

Cutting off incisors of great length in a young horse need not be considered a fraudulent act on the part of the dealer, for, while shortening of the long teeth of old age is a trick to deceive the ignorant, who associates long teeth with great age, the shortening of the teeth of a young horse gives a table indicating a greater age, and will not deceive the expert, who should always examine any horse on purchase.

EXCESS OF LENGTH OF THE INCISORS OF THE UPPER JAW.

This anomaly constitutes what is commonly known as *parrot-mouth*, (Fig. 69, 69*a*), on account of the analogy in the appearance of the upper jaw, to the corresponding beak of this bird. The upper teeth may acquire a length of 2 1-2 inches, and are frequently very much curved forward and downward, while their posterior faces are worn away to a sharp bevel by their contact with the inferior incisors; these latter are frequently shorter than normal, and the parrot's beak is formed by the pincher and intermediate teeth with only the internal border of the corner teeth,

FIF. 69*a*.

while the rest of the latter is worn into a deep notch. These deformities are frequently much greater on one side of the jaw than on the other.

In some horses five years old, the upper jaw projects a line or two in front of the inferior jaw, whilst the posterior surface of the teeth in both jaws corresponds, which produces excessive wearing of the posterior part of the upper incisors, leaving a little line in front of their table, which predisposes to the formation of parrot-mouth. Usually, however, parrot-mouth is only seen in very old horses. It interferes more or less, according to its development, in the prehension of food, especially with oats, as the projection of the teeth interferes somewhat with the movements of the lips; the elongated teeth also render the maximum extent, to which the incisors can open, less than in a normal mouth; the latter can usually open 2 1-2 inches, or a little more, while in certain parrot-mouths the space between the open incisors, is only 1 1-4 to 1 1-2 inches. The bevelled and the distorted tables of the incisors in a parrot-mouth, render the determination of age, for these, practically impossible. The judgment of age may be based upon the relative characters, as to the inclination of the teeth, their color, their size, and what one supposes would be their tables, were they cut to their proper length. It frequently happens that a parrot-mouth so interferes with mastication on the part of the animal, that it becomes necessary to resort to operative measures, with the saw and file, to give the animal a normal mouth. This operation not only relieves the animal, but restores it to the appearance of its real age, and does not enter into the category of fraudulent measures.

### EXCESSIVE LENGTH OF SINGLE TEETH OF THE JAW.

Mouths occasionally are seen in which only one, two, or a small number of the teeth are distorted, this occurs at times on one side only, or it occurs frequently that the teeth on one side, are very long, and those on the opposite side very short, corresponding with the inverse condition in the other jaw. In cases like this, as should always be the rule, as is necessary in all horses to determine age, to inspect them from both sides; in horses of seven or eight years old, a slight increase of wearing on one side of the jaw, will frequently make in the table mark, a deviation of a year or two in the appearance of the animal's age, which is readily corrected by a second examination from the other side.

DEFICIENCY OF LENGTH OF THE FREE PORTION OF THE INCISORS.

Deficiency of length is seen only in very old horses, the teeth are always in apposition, except in the case of cribbing horses, when they may be so worn off so as not to reach those of the

FIG. 70.

other jaw. The diminution in length may be so great, that nothing but a mere stub remains; this condition is frequently accompanied by *cementoma* or tumors thrown out by the irritated alveolar periosteum.

[TO BE CONTINUED.]

## Figures and Tables

**Fig. 69. f1:**
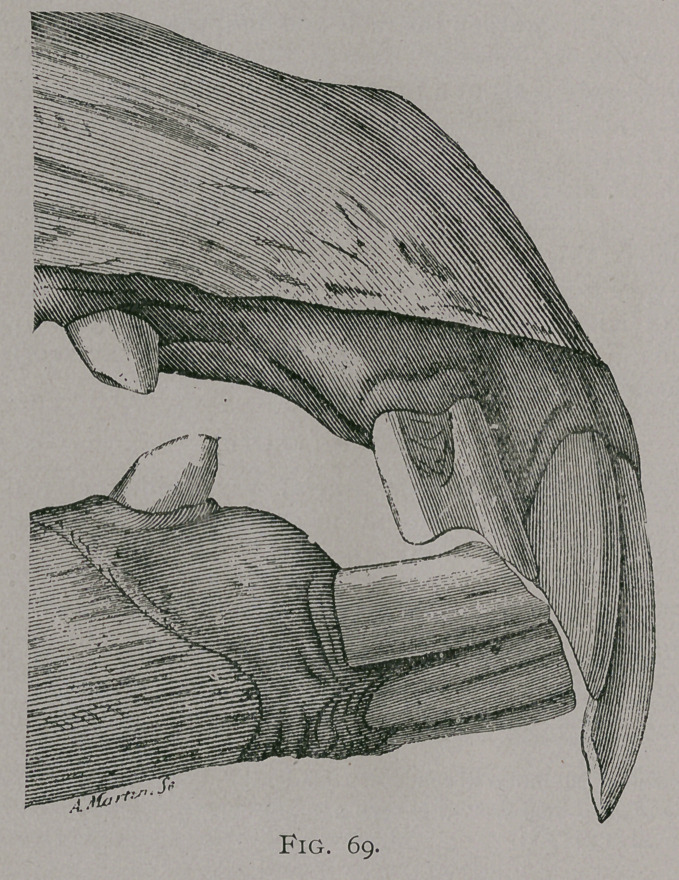


**Fig. 69a. f2:**
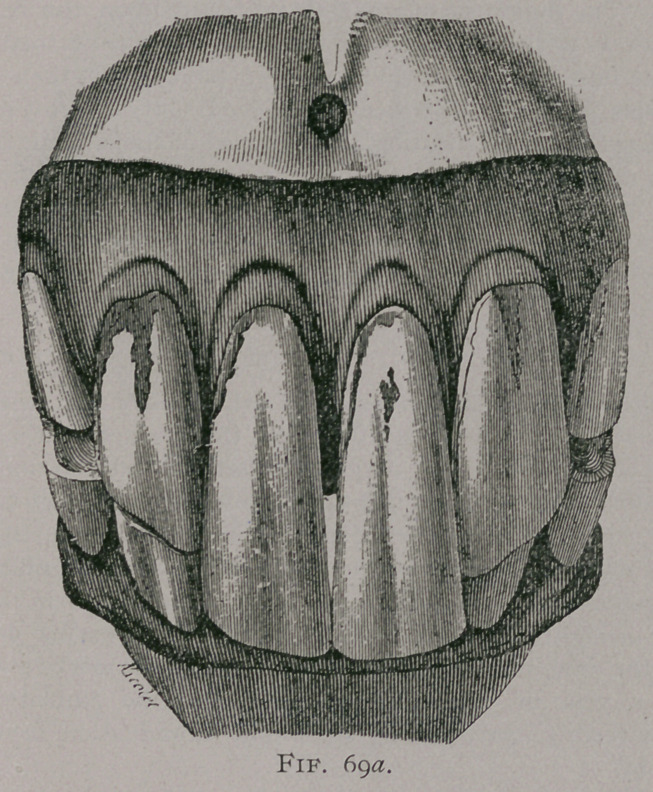


**Fig. 70. f3:**